# Model-Based Electroencephalogram Instantaneous Frequency Tracking: Application in Automated Sleep–Wake Stage Classification

**DOI:** 10.3390/s24247881

**Published:** 2024-12-10

**Authors:** Masoud Nateghi, Mahdi Rahbar Alam, Hossein Amiri, Samaneh Nasiri, Reza Sameni

**Affiliations:** 1 Department of Biomedical Informatics, School of Medicine, Emory University, Atlanta, GA 30322, USA; hossein.amiri@emory.edu (H.A.); snasiri@dbmi.emory.edu (S.N.); 2 Independent Researcher, Shiraz 7197688711, Iran; m.rahbaralam@gmail.com; 3 Department of Biomedical Engineering, Georgia Institute of Technology, Atlanta, GA 30332, USA

**Keywords:** automatic sleep staging, electroencephalogram, instantaneous frequency tracking, Kalman filter

## Abstract

Understanding sleep stages is crucial for diagnosing sleep disorders, developing treatments, and studying sleep’s impact on overall health. With the growing availability of affordable brain monitoring devices, the volume of collected brain data has increased significantly. However, analyzing these data, particularly when using the gold standard multi-lead electroencephalogram (EEG), remains resource-intensive and time-consuming. To address this challenge, automated brain monitoring has emerged as a crucial solution for cost-effective and efficient EEG data analysis. A critical component of sleep analysis is detecting transitions between wakefulness and sleep states. These transitions offer valuable insights into sleep quality and quantity, essential for diagnosing sleep disorders, designing effective interventions, enhancing overall health and well-being, and studying sleep’s effects on cognitive function, mood, and physical performance. This study presents a novel EEG feature extraction pipeline for the accurate classification of various wake and sleep stages. We propose a noise-robust model-based Kalman filtering (KF) approach to track changes in a time-varying auto-regressive model (TVAR) applied to EEG data during different wake and sleep stages. Our approach involves extracting features, including instantaneous frequency and instantaneous power from EEG, and implementing a two-step classifier for sleep staging. The first step classifies data into wake, REM, and non-REM categories, while the second step further classifies non-REM data into N1, N2, and N3 stages. Evaluation on the extended Sleep-EDF dataset (Sleep-EDFx), with 153 EEG recordings from 78 subjects, demonstrated compelling results with classifiers including Logistic Regression, Support Vector Machines, Extreme Gradient Boosting (XGBoost), and Light Gradient Boosting Machine (LGBM). The best performance was achieved with the LGBM and XGBoost classifiers, yielding an overall accuracy of over 77%, a macro-averaged F1 score of 0.69, and a Cohen’s kappa of 0.68, highlighting the efficacy of the proposed method with a remarkably compact and interpretable feature set.

## 1. Introduction

Quality-sleep is crucial for physical and mental well-being, influencing memory consolidation, emotional regulation, and cognitive function [1]. However, sleep quality and duration vary significantly among individuals due to factors such as lifestyle, age, and medical conditions. Recognizing transitions between wakefulness and different sleep stages is essential, as these transitions provide insights into sleep architecture and help in diagnosing and managing sleep disorders. Conditions like sleep apnea and narcolepsy, for example, often present abnormal sleep–wake patterns, affecting overall health and quality of life.

The current gold standard for sleep staging involves manually analyzing polysomnography (PSG) recordings according to the American Academy of Sleep Medicine (AASM) guidelines [2]. These guidelines classify each 30-second epoch into Wake (W), Rapid Eye Movement (REM), and Non-REM stages one to three (N1, N2, N3). However, this method is labor-intensive, time-consuming, and subject to significant inter-expert variability [3,4]. The five-stage classification is a human construct intended to simplify interpretation, but this imprecision can lead to varying levels of agreement among raters, with international agreement rates as low as 60% and slightly over 80% within the same institution [5]. Therefore, AI-based automated sleep staging is viewed as a promising approach for achieving more objective and consistent results.

Automated systems are also expected to play a crucial role in large-scale epidemiological research linking sleep patterns to health and wellness. Sleep studies measure “duration,” or the time spent in different sleep stages (e.g., REM, N1, N2, N3), with deviations potentially indicating disorders such as insomnia, neurodegenerative diseases, REM sleep behavior disorder, or Parkinson’s disease [6,7,8,9]. “Frequency” refers to the transitions between sleep stages throughout the night. Normal sleep involves predictable cycling through these stages multiple times, and changes in the frequency of these transitions can reveal insights into sleep continuity and quality. Frequent awakenings, for example, may suggest disturbances or disorders [10].

Both duration and frequency are essential for understanding sleep patterns, identifying abnormalities, and guiding diagnosis and treatment. These metrics assess sleep quality, detect disorders like sleep apnea or insomnia, and tailor interventions for improved sleep health [6,11].

The field of sleep EEG analysis involves a variety of features and classification methods that enhance our understanding of sleep patterns and disorders. Key features used in this domain include energy features, Fourier transform coefficients [12,13], wavelet coefficients [14,15,16,17], entropic features [13,18], and fractal features [19]. These features are crucial for various classification techniques applied in sleep EEG analysis, such as discriminant analysis [18,19], hidden Markov models [12,20,21], neural networks [15,16,17,22,23,24,25,26], random forests [18,27], and support vector machines [28,29,30,31,32].

Spectral features, which capture essential information about the EEG’s power spectral density (PSD), have become fundamental in sleep EEG analysis [33]. PSD is highly correlated with distinct neuronal activity frequency bands, making it a valuable tool. Methods typically assume EEG stationarity during the estimation period to estimate EEG PSD. However, the EEG time interval length is crucial, as it affects the trade-off between time and frequency resolution. Achieving a balance that exceeds the Heisenberg–Gabor lower bound can be challenging [34]. While PSD is useful, understanding sleep stages requires deeper insights into the dynamics of frequency components, which may not be evident from PSD alone. Thus, tracking the dominant instantaneous frequency (IF) of EEG signals over time is important. This method aligns with the dynamic nature of neuronal oscillations during sleep stages, as neurons exhibit specific rhythmic patterns in different sleep phases that are reflected in the EEG [35].

The use of auto-regressive moving-average (ARMA) models for parametric model root tracking in EEG analysis, utilizing a recursive least squares (RLS) algorithm, was first introduced by Patomäki et al. [36]. It has been suggested that the displacement of poles in an auto-regressive (AR) model fitted to nonstationary EEG data carries physiological significance [36,37,38]. Furthermore, IF has been found to exhibit the strongest correlation with changes in the level of consciousness, particularly in the detection of depth of anesthesia (DOA) [39].

Despite advancements in sleep stage classification, existing methods face significant challenges. Traditional human-based annotations, while accurate, are resource-intensive, subject to inter-rater variability, and time-consuming, limiting their scalability. Automated classification methods offer a promising alternative, but often fall short in capturing the fine-grained, time-frequency characteristics of the EEG that reflect the dynamic nature of neuronal oscillations. These automated methods also tend to struggle with balancing time and frequency resolution effectively, which is essential for identifying transitions across sleep stages. Traditional time-frequency analysis methods for IF extraction, such as spectrograms, scalograms, Wigner–Ville distributions, and Hilbert transforms, each have limitations when applied to sleep EEG analysis. Fourier transform-based methods assume signal stationarity and face an inherent time-frequency resolution trade-off. Time-scale (wavelet) methods, while better at handling non-stationarity, still fall short in precise frequency localization at higher frequencies. Other time-frequency-based methods, like the Wigner–Ville distribution, can provide better resolution but suffer from cross-term interference when analyzing multi-component signals like EEG. The Hilbert transform, though widely used for instantaneous frequency estimation, is highly sensitive to noise and background cerebral activity, often leading to unstable frequency estimates in EEG data.

To address these limitations, we propose a robust approach that combines a refined feature set with conventional classifiers enhanced by a model-based Kalman filtering framework for EEG frequency tracking. Our method tracks the IF and envelope of EEG data within key frequency bands (delta, theta, alpha, beta, and gamma), leveraging the dynamic properties of EEG rhythms as core features for sleep stage classification. The proposed Kalman filter-based method offers precise IF tracking across EEG sub-bands with confidence intervals. We demonstrate that the proposed Kalman filtering method offers several key advantages for this application: it is the optimal state estimator in the presence of noise; naturally handles the non-stationary nature of EEG through its recursive updating mechanism, inherently offers statistical confidence bounds for its estimates, and can track rapid frequency changes without the time-frequency resolution limitations of stationary transform-based methods. This approach is particularly effective because the IF of the EEG closely aligns with physiological patterns recognized by sleep experts, this method can improve the reliability of automated sleep staging systems for applications in sleep research and clinical diagnostics.

## 2. A Kalman Filter-Based IF Estimation Scheme

This section provides a detailed explanation of the methodology used to extract IE and IF features from individual EEG channels and frequency bands.

### 2.1. The Concept of IE and IF

In non-stationary signals like EEG, both amplitude and frequency vary over time. To accurately characterize these variations, it is crucial to estimate their instantaneous fluctuations. The IE and IF of a signal can be extracted using its analytic form [40]. Let $y_{n}^{a}$ represent the analytic form of a narrow-band discrete-time signal $y_{n}$ with sampling frequency $f_{s}$,

(1) $$y_{n}^{a}=y_{n}+j{\tilde{y}}_{n}=A_{n}e^{j\phi_{n}}$$

where ${\tilde{y}}_{n}$ denotes the Hilbert transform of $y_{n}$, $A_{n}$ represents the modulus of the analytic form, known as the IE, and $\phi_{n}$ is the *instantaneous phase* (IP) of $y_{n}^{a}$ in radians.

The IF is the scaled time derivative of the instantaneous phase (IP). For discrete-time signals and at low frequencies (relative to the sampling frequency), the IF can be approximated by the normalized first-order difference of the IP, scaled to the Nyquist range $\left[0,f_{s}\right)$,

(2) $$f_{n}=f_{s}\frac{\phi_{n}-\phi_{n-1}}{2\pi }$$

The estimation of EEG IF and IE is notably affected by the level of background cerebral activity [41]. In [42,43,44], a method for performing these parameter estimations effectively within a robust statistical framework was presented. In this context, we utilize a robust extension of the IF estimation algorithm from prior works [38,45]. This extension incorporates a variable dynamic model and considers the specific implications of tracking both IF and IE features for our application of interest.

### 2.2. Bandpass Filtering

The concept of IF relies on the assumption of a dominant frequency peak at each time instant [46]. However, extracting IF from time-varying, multi-component signals like EEG poses significant challenges. These challenges are due to several factors: the inherent complexity of EEG signals, which contain overlapping frequency components that vary over time; their non-stationary nature, with fluctuating frequency content; the occurrence of frequency mixing, which can introduce artifacts or blending that obscure the dominant IF; limitations in resolution, which impacts the precise tracking of rapid frequency changes, especially in the low-frequency ranges; and the high susceptibility of EEG to noise and artifacts.

To mitigate these issues, a common strategy is to apply narrow-band band-pass filtering prior to feature extraction. To maintain the inter-band time lags, we use a zero-phase forward-backward finite impulse response (FIR) filter, with lower and upper cutoff frequencies $f_{l}$ and $f_{h}$, respectively. After filtering, frequency tracking is performed across different EEG frequency bands—delta, theta, alpha, beta, and gamma—to monitor IF variations within these key subbands simultaneously.

### 2.3. Amplitude Normalization

In a previous study [45], amplitude normalization by the EEG modulus was used to reduce frequency estimation bias,

(3) $${\bar{y}}_{n}=\frac{y_{n}}{A_{n}}$$

where $A_{n}$ is the analytical form modulus (IE) of the EEG defined in (1).

This procedure significantly improved the accuracy of estimating both the modulus and phase of the TVAR filter response. In this work, we apply this step prior to our model-based Kalman filter-based scheme for robust instantaneous frequency estimation.

### 2.4. Time-Varying Auto-Regressive (TVAR) EEG Instantaneous Frequency Modeling

Various techniques have been employed for estimating IF in non-stationary signals, which can be broadly categorized into parametric and non-parametric methods. Non-parametric methods include the short-time Fourier transform (STFT) [47,48], the Hilbert transform [49], wavelet-based algorithms [34], and Wigner–Ville distributions [50], which as detailed in the introduction, have fundamental limitations in analyzing non-stationary signals like the EEG.

In contrast, parametric methods use linear or nonlinear models based on the signal’s complexity. Auto-regressive (AR) and auto-regressive moving average (ARMA) models are common linear parametric models applied to biomedical signals. The time-varying auto-regressive (TVAR) model, an extension of the AR model with variable parameters, is particularly well-suited for non-stationary signals like EEG, which exhibit time-varying spectral properties [51]. By incorporating this TVAR model within a Kalman filtering framework, we can overcome the limitations of non-parametric methods by adaptively tracking time-varying frequency components, thereby improving the time-frequency resolution trade-off.

In the AR model, a stationary discrete-time signal $y_{n}$ is represented as the output of a linear time-invariant system with white noise input,

(4) $$y_{n}=\sum_{k=1}^{p}c_{k}y_{n-k}+v_{n}$$

where $c_{k}$ (k=1,…,p) are AR model coefficients, $v_{n}$∼$N(0,r_{n})$ is zero-mean white Gaussian noise (WGN), and *p* is the order of the AR model. Accordingly, the AR process is equivalent to applying WGN to the following filter in the frequency domain:

(5) $$H\left(z\right)=\frac{1}{1-\sum_{k=1}^{p}c_{k}z^{-k}}$$

where the configuration of poles in the frequency domain—including their number, radius, and angle—determines the spectral characteristics of the generated stochastic process. Therefore, the number of oscillatory components is governed by the order of the AR model, denoted by *p*. For example, a second-order AR model (AR(2)) indicates a single dominant frequency component. Generally, an AR(2) model can act as a low-pass, band-pass, or high-pass filter, depending on the pole locations. The filter’s bandwidth (bw) and center frequency ($\omega_{0}$) are determined by the pole radius (*r*) and pole angle (ϕ), respectively.

**Figure 1:**
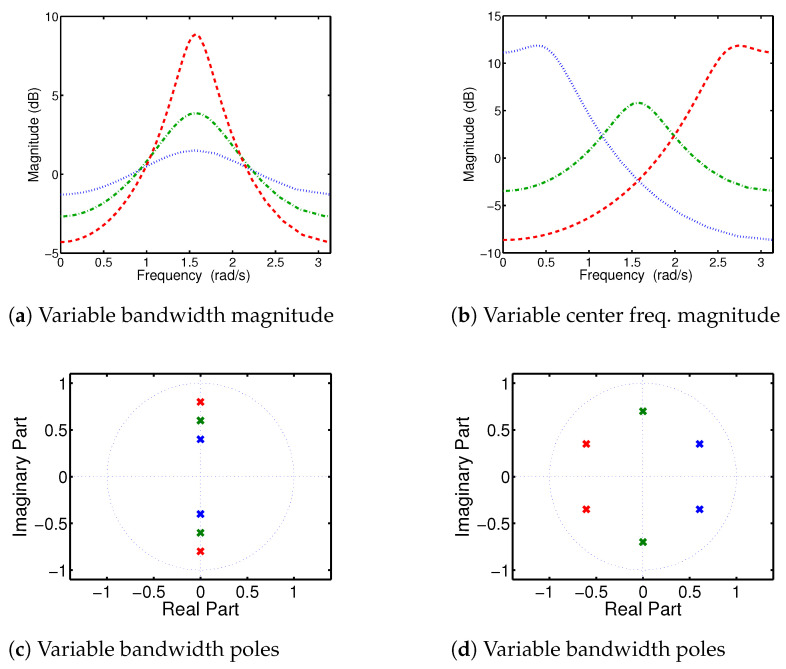
Auto-regressive model pole locations vs. AR(2) spectral characteristics. (**a**,**c**): As the pole moves closer to the origin (from red to green to blue), the bandwidth of the corresponding band-pass filter increases. (**b**,**d**): The pole locations on the z-plane determine the filter type: high-pass (red), band-pass (green), and low-pass (blue).

The relationship between the location of AR(2) complex conjugate poles in the z-plane and the parameters of the corresponding filter $\left(bw,\omega_{0}\right)$ is illustrated in Figure 1. The magnitude response $\left|H\left(e^{j\omega }\right)\right|$ demonstrates that as the pole radius approaches the unit circle, the filter bandwidth (bw) decreases, eventually resulting in a narrow-band filter that exhibits resonance at the central radian frequency $\omega_{0}$.

In non-stationary signals like EEG, the frequency response H(z) varies over time. However, presumably the movement of the poles—and the corresponding changes in the transfer function—typically occurs relatively slowly. The frequency response of a time-varying autoregressive model of order two, TVAR(2), with slowly varying coefficients (having negligible variations across the data windows of interest), can be expressed as follows:

(6) $$H\left(z;n\right)=\frac{1}{1-c_{1}\left(n\right)z^{-1}-c_{2}\left(n\right)z^{-2}}$$

We can show that the squared magnitude response of this TVAR(2) model is $|H\left(e^{j\omega };n\right){|}^{2}={\left[\left(1+c_{1}{\left(n\right)}^{2}+c_{2}{\left(n\right)}^{2}\right)+2c_{1}\left(n\right)\left(c_{2}\left(n\right)-1\right)\cos\left(\omega \right)-2c_{2}\left(n\right)\cos\left(2\omega \right)\right]}^{-1}$, with its peak at

(7) $$\omega_{0}\left(n\right)=\arccos\left(\frac{c_{1}\left(n\right)\left[c_{2}\left(n\right)-1\right]}{4c_{2}\left(n\right)}\right)$$

as demonstrated in Figure 1. The magnitude of the poles is related to the stability condition of the resulting stochastic process [52]. The following conditions guarantee the stability of the causal system’s output ([53] Chapter 3.1):

(8) $$c_{2}\left(n\right)\pm c_{1}\left(n\right)<1,\;\mathrm{and}\;\left|c_{2}\left(n\right)\right|<1$$

In EEG data, the time-varying normalized center frequency $\omega_{0}\left(n\right)$ is associated with the frequency at which most cortical neurons in the vicinity of the recording site oscillate in synchrony [38]. The dominant EEG frequency manifests as local peaks in the EEG spectrum. In our TVAR(2) model, the dominant instantaneous frequency of the EEG, denoted by IF(n), can be associated with $\omega_{0}\left(n\right)$ as follows:

(9) $$\mathrm{IF}\left(n\right)=\frac{f_{S}}{2\pi }\omega_{0}\left(n\right)$$

Note that the choice of TVAR(2) in this context is motivated by its ability to track a single dominant frequency component. A second-order model provides sufficient degrees of freedom to track the dominant frequency and bandwidth (or Q-factor) while maintaining model simplicity and computational efficiency. As shown in Equations (7) and (8), the TVAR(2) model parameters are directly related to the instantaneous frequency and effective bandwidth, providing a clear physiological interpretation of the model parameters. While higher-order models could better represent the EEG spectra, they also introduce additional poles that might capture spurious frequency components or noise, potentially degrading the tracking of the primary frequency component of interest within each band. The dominant frequency paradigm modeled by TVAR(2) also aligns with the physiological understanding that each EEG sub-band primarily contains a single dominant oscillatory component that sleep specialists associate with the level of awareness.

### 2.5. TVAR Model Parameter Tracking Using Kalman Filter

The Kalman filter (KF) has been widely used for tracking and estimating TVAR coefficients in the literature [45,54,55]. The linear Kalman filter, under the Gaussian process and measurement noise assumption, is an optimal minimum mean square error (MMSE) estimator [56]. The KF literature and its optimization techniques are very well established, making them particularly well-suited for EEG instantaneous frequency tracking.

Using a KF framework, the outline of our proposed framework is as follows: we model the EEG in different sub-bands with a TVAR(2) model. We use a Kalman filter to track the instantaneous frequency of the EEG in a robust manner and with statistical confidence intervals. As a case study, these frequencies are next used for sleep staging classification.

In the context of a TVAR(2) model, if we consider the coefficient vector $c_{n}={\left(c_{1}\left(n\right),c_{2}\left(n\right)\right)}^{T}$ as the system’s state vector and $y_{n}$ as the narrow-band EEG in a specific frequency band, the TVAR(2) model can be represented in the following state-space form:

(10) $$c_{n+1}=c_{n}+w_{n}$$

(11) $$y_{n}=h_{n}^{T}c_{n}+v_{n}$$

where $v_{n}$∼$N(0,r_{n})$ is a zero-mean white Gaussian observation noise with variance $r_{n}$, and $h_{n}={\left(y_{n-1},y_{n-2}\right)}^{T}$ consists of the two preceding observations according to AR model in (4). According to the state evolution model (10), the coefficient vector evolution has been assumed to follow a first-order AR model in the form of a Random Walk or Wiener process with Gaussian process noise vector $w_{n}$ and a diagonal covariance matrix $Q_{n}\overset{\Delta }{=}\mathrm{diag}\left(\left[q_{n}^{1},q_{n}^{2}\right]\right)$, which is a common technique in AR modeling [57,58]. Accordingly, the rate of AR parameter evolution is controlled by the covariance matrix entries $q_{n}^{1}$, $q_{n}^{2}$, which we leave as hyperparameters to be optimized.

The forward KF equations for sequential estimation of the state vector are as follows:

(12) $${\hat{c}}_{n+1}^{-}={\hat{c}}_{n}^{+}$$

(13) $$P_{n+1}^{-}=P_{n}^{+}+Q_{n}$$

(14) $$k_{n}=\frac{P_{n}^{-}h_{n}}{h_{n}^{T}P_{n}^{-}h_{n}+r_{n}}$$

(15) $$e_{n}=y_{n}-h_{n}^{T}{\hat{c}}_{n}^{-}$$

(16) $${\hat{c}}_{n}^{+}={\hat{c}}_{n}^{-}+k_{n}e_{n}$$

(17) $$P_{n}^{+}=\left(I-k_{n}h_{n}^{T}\right)P_{n}^{-}{\left(I-k_{n}h_{n}\right)}^{T}+k_{n}r_{n}k_{n}^{T}$$

where $P_{n}\in R^{2\times 2}$ is the state estimation error covariance matrix, $I\in R^{2\times 2}$ is the identity matrix, $k_{n}$ is the *Kalman gain* vector, and $e_{n}$ is the error in observation prediction, known as the *innovation signal*. In all equations, the superscripts − and + refer to the estimation of the corresponding quantity before and after observation arrival, respectively (also known as priors and posteriors). We have used the Joseph stabilized form of the state vector covariance matrix update in (17), which ensures that the covariance update remains positive semi-definite [59].

The recursion is initialized by ${\hat{c}}_{0}^{-}=\mu_{0}$ and $P_{0}^{-}=P_{0}$ where $\mu_{0}={\left(\mu_{1},\mu_{2}\right)}^{T}$ and $P_{0}\in R^{2\times 2}$ are presumed mean and covariance of the initial Gaussian state vector $c_{0}$∼$N(\mu_{0},P_{0})$. The initial state vector can be set by applying the Yule–Walker method on the entire signal offline. A stable KF is not sensitive to the choice of the initial condition, as its impact vanishes over time. Practically, it is recommended to overestimate $P_{0}$ (for example, by one or two orders of magnitude greater than their expected range) to enable the KF to adapt them over time.

The KF is causal, as it uses the two previous observations to estimate the current state. For offline processing, better performance can be obtained by using a noncausal Kalman Smoother (KS). The KS basically consists of a forward KF followed by a backward recursive smoothing stage. Depending on the smoothing strategy, smoothing algorithms are usually classified into *fixed-lag* or *fixed-interval* [60,61]. Herein, we use a fixed-interval KS for the offline sleep staging algorithm. In this scheme, having the forward estimated states and their covariance matrix from sample n=1 to n=N, the backward estimation process is applied recursively for n=N−1 to n=1 as follows:

(18) $${\hat{c}}_{n|N}={\hat{c}}_{n}^{+}+S_{n}\left({\hat{c}}_{n+1|N}-{\hat{c}}_{n+1}^{-}\right)$$

(19) $$P_{n|N}=P_{n}^{+}+S_{n}\left(P_{n+1|N}-P_{n+1}^{-}\right)S_{n}^{T}$$

where $S_{n}\overset{\Delta }{=}P_{n}^{+}{\left[P_{n+1}^{-}\right]}^{-1}$. In these equations, n|N denotes *n*-th smoothed version of the state vector or covariance matrix, using *N* samples of the same quantity. This implementation of the KS is known as the Rauch–Tung–Striebel two-pass KS algorithm [60].

### 2.6. KF Parameter Selection and Optimization

It can be shown that for the dynamic model (10) and (11), the KF equations are only a function of the ration $\rho_{n}=\mathrm{diag}\left(Q_{n}\right)/r_{n}=\left[q_{n}^{1}/r_{n},q_{n}^{2}/r_{n}\right]$ [60]. Therefore, one only needs to tune $\rho_{n}$ instead of both parameters $Q_{n}$ and $r_{n}$. In order to set this ratio, we use the running average of the actual innovation process variance ($e_{n}^{2}$) over the presumed innovation process covariance $h_{n}^{T}P_{n}^{-}h_{n}+r_{n}$, over a sliding window of length *L*,

(20) $$\lambda_{n}=\frac{1}{L}\sum_{k=n-L+1}^{n}\frac{e_{k}^{2}}{h_{k}^{T}P_{k}^{-}h_{k}+r_{k}}$$

If the KF parameters $Q_{n}$ and $r_{n}$ (or the ratio $\rho_{n}$) are selected correctly, $\lambda_{n}$ fluctuates around unity [61,62]. In the current case, $\rho_{n}$ has been selected such that $\lambda_{n}$ fluctuates around 1 in the awake state. In real sleep studies, this procedure can be performed per subject by a short learning process before sleep or by adaptively updating $r_{n}$ or $Q_{n}$ to maintain $\lambda_{n}$ around 1, which is a classical technique in KF engineering ([63] Ch. 6), ([64] Ch. 8). Our analysis of the $\lambda_{n}$ parameter shows that after approximately 30 s, $\lambda_{n}$ consistently fluctuates around unity, indicating a consistent Kalman filter performance.

## 3. Evaluation

### 3.1. Dataset

**Figure 2:**
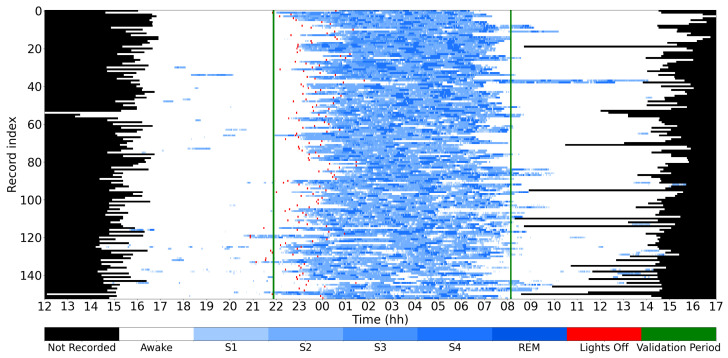
Sleep stage patterns of all records of the Sleep-EDFx Dataset [65,66], aligned on a time axis from 12 PM to 5 PM the following day. Green lines denote segments selected for model validation. Red bars indicate times when lights were turned off. Blue and white segments represent sleep and wake periods, respectively. Due to varying recording lengths and start times across recordings, unrecorded periods are denoted in black.

We showcase the application of the proposed EEG frequency tracking scheme for sleep staging. We use two EEG channels, Fpz–Cz and Pz–Oz, and the electrooculogram (EOG) channel sampled at 100 Hz, from the extended Sleep-EDF dataset (Sleep-EDFx), as detailed in [65,66]. This dataset comprises PSG recordings from 153 sleep cassettes collected from 78 healthy subjects (36 males and 41 females), ranging in age from 25 to 101 years. Each subject PSG was collected across two consecutive day–night periods (except for 14 subjects who had data from only one night), with hypnogram annotations provided for 30 s intervals. The hypnogram labels include “W” (wakefulness), “S1”, “S2”, “S3”, and “S4” (non-REM sleep), “R” (REM sleep), “M” (movement time), and “?” (unscored segments). Movement times and unscored segments, being negligible compared to the labeled epochs, were omitted from the analysis. This aligns with the latest AASM standard, where “movement times” have been excluded from sleep staging labels [67]. All hypnograms were manually scored by trained technicians following the Rechtschaffen and Kales guideline [68], but using Fpz–Cz/Pz–Oz EEGs instead of C4–A1/C3–A2 EEGs, as recommended in [69]. The subject-wise sleep stage labels of this dataset are illustrated in Figure 2.

To assess the adaptability of the classification algorithms to the AASM standard, S3 and S4 were combined into a single Non-REM stage (N3). However, other AASM parameters could not be met with the current dataset due to differences in scoring rules compared to the Rechtschaffen and Kales (R&K) standard [68].

**Figure 3:**
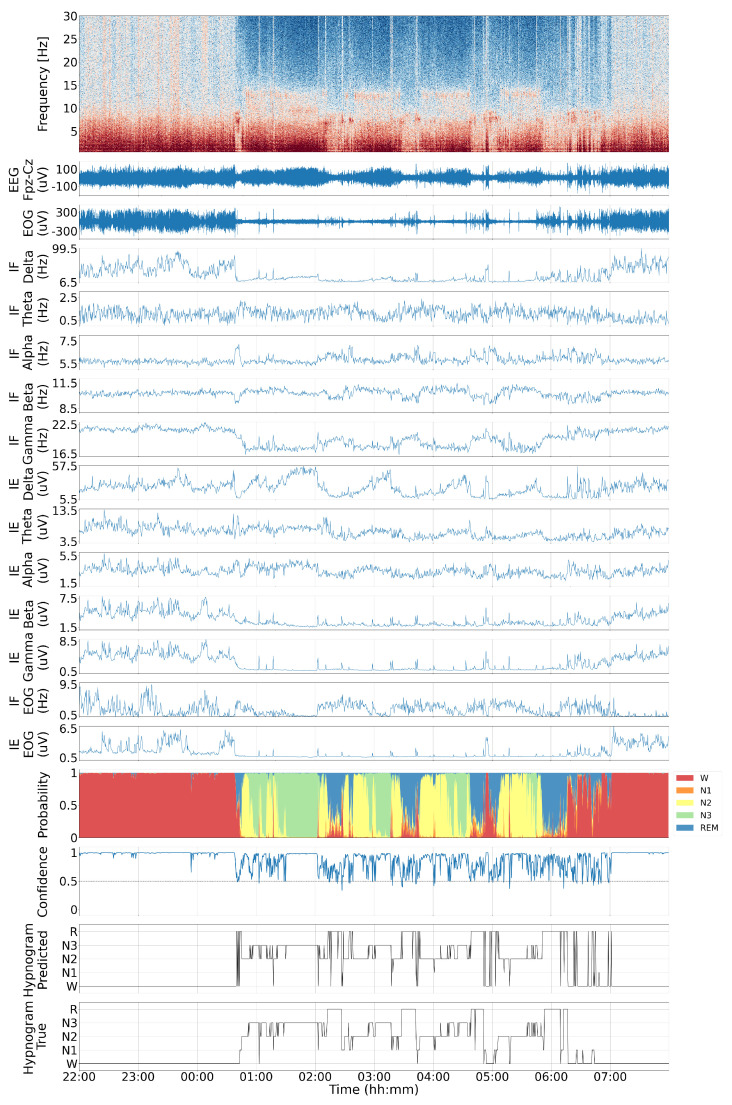
Spectrogram of EEG signal from channel Fpz-Cz, along with the EEG signal, EOG signal, estimated IF and IE of different sub-bands of EEG signal, estimated IF and IE of the EOG signal, output probabilities of different classes, the confidence of the model in classification [71], and the predicted and true hypnogram labels for record SC4001 of Sleep-EDFx.

Previous studies have highlighted a proportional relationship between changes in consciousness levels and variations in energy across different frequency bands [39,70]. To demonstrate this, the spectrogram of a ten-hour sample of wakefulness and sleep EEG recorded from the Fpz–Cz channel, along with its corresponding hypnogram, is shown in the first and last plots of Figure 3. The wide-band spectrogram in Figure 3 shows that the sleep state mainly manifests in the energy of alpha and beta rhythms. To validate this observation more rigorously, we divided the wide-band frequency range of the EEG into sub-bands corresponding to common brain rhythms between 0.5 and 50 Hz and applied the KF-based IP/IF extraction algorithm across frequency bands in parallel, as detailed below.

### 3.2. Implementation

The developed algorithms and machine learning models were all implemented in Python 3.11.9 and executed on a Linux-based high-performance computing cluster (using CPU resources) in the Department of Biomedical Informatics, Emory University, Georgia, USA. The data processing pipeline and machine learning models were developed using Python’s scientific computing libraries, including NumPy 1.26.4 and SciPy 1.14.1 for signal processing, and scikit-learn 1.5.2 for machine learning implementations. The LGBM 4.5.0 and XGBoost 2.1.1 models were implemented using their respective Python packages.

### 3.3. Processing and Feature Extraction Pipeline

**Figure 4:**
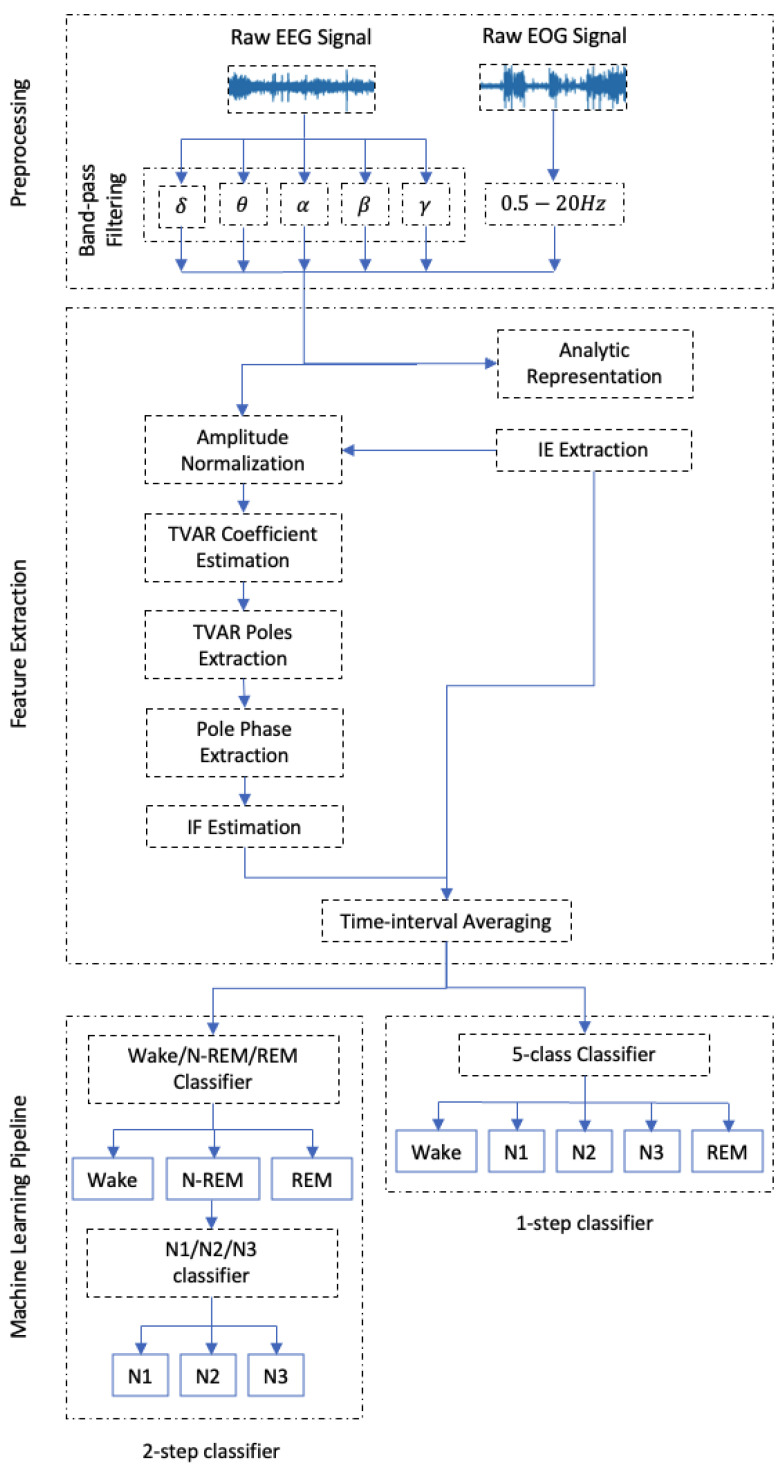
Sleep staging processing pipeline. The diagram depicts the process from raw signal pre-processing, including band-pass filtering, through feature extraction using Kalman filter-based time-varying AR instantaneous frequency tracking, and envelope estimation. The features are provided to the machine learning classification stages, to identify various sleep states (Wake, Non-REM, REM) and sub-stages (N1, N2, N3).

The proposed algorithm was applied to the Fpz–Cz and Pz–Oz EEG channels, as well as the EOG channel. Figure 4 presents an overview of the feature extraction and analysis algorithm, consisting of the following steps:

1. In the pre-processing stage, the raw signal is passed through five parallel FIR band-pass filters with bandwidth ranges corresponding to the δ (0.5–4.0 Hz), θ (4.0–8 Hz), α (8.0–13.0 Hz), β (13.0–30.0 Hz), and γ (30–50 Hz) brain rhythms. The EOG channel is separately processed using a band-pass filter with a range of 0.5–20 Hz to preserve its main frequency components.
2. The EEG and EOG of each subband are represented in analytic form (1), and their modulus are set as the IE.
3. Signals are normalized by their analytical form modulus using (1).
4. The IF of the normalized signals are estimated using the robust KF algorithm detailed in Section 2.
5. Steps 2 and 4 generate five pairs of IF-IE for each EEG channel and a single pair of IF-IE for the EOG channel, all of which match the original signals in length. As hypnogram labels are provided for 30 s intervals, time-interval averaging is applied to IF-IE vectors to compute the mean IE-IF over non-overlapping 30 s windows.

The generated feature vectors from the different frequency bands of all channels, together with their hypnogram labels, are saved for classification and feature importance analysis.

### 3.4. Classification Pipeline

We assess the effectiveness of the proposed EEG feature extraction and tracking scheme for classification of EEG sleep stages with the hypnogram labels.

The processing pipeline in Section 3.3 results in a total of 22 features for each subject per each 30 s epoch.

As illustrated in Figure 4, the machine learning pipeline consists of two steps. First, a classifier is trained to distinguish between awake, non-REM, and REM stages. Next, a second classifier is used to further differentiate between N1, N2, and N3 stages within the non-REM category. Shapley Additive Explanations (SHAP) values are computed at each step to identify the key features for distinguishing sleep stages. These results are compared with those from a single-step classifier that directly categorizes the data into five sleep stages.

We considered four classifiers: Logistic Regression (LR), Support Vector Machine (SVM) with a linear kernel, Extreme Gradient Boosting (XGBoost), and Light Gradient Boosting Machine (LGBM). To address class imbalance in the first step (Wake/REM/NREM classification), initial weights were based on typical sleep stage proportions in healthy adults: 75% non-REM (5% for N1, 45% for N2, 25% for N3), and 25% for REM [72]. These proportions were also used in our one-step classifier. In the second-step classifier (N1/N2/N3 classification), which processes only non-REM stages, weights were adjusted to reflect the distribution within non-REM stages only: 20% for N1, 66% for N2, and 14% for N3, based on the relative proportions in our dataset (16%, 53%, and 11%, respectively).

The classifiers were validated using subject-wise 10-fold cross-validation (CV). To prevent data leakage, feature sets for training and testing were separated by subject, ensuring no participant’s data appeared in both sets. In each fold, 10% of participants (8 out of 78 subjects) were allocated to the test set so that each subject appeared exactly once in the test set. While the models were trained on complete recordings from the training subjects, validation was performed specifically on the 10 PM to 8 AM period to simulate realistic nighttime conditions. This validation window was chosen based on the documented lights-off times and the approximately 24-h duration of each recording. Figure 2 shows the distribution of different sleep stages for all subjects within this time frame, confirming that the testing interval is appropriate and covers the main distribution of sleep stages.

The classification performance is assessed using the classification confusion matrix. As a measure of overall performance for each subject, we also report Cohen’s kappa (κ) and the macro-averaged F1 score to evaluate the robustness of the classifiers.

## 4. Results

In this section, we present the results of applying the detailed algorithm through selected visual inspections followed by the overall quantitative performance assessment across all records.

### 4.1. Visual Inspection

A typical sample of the estimated IF using TVAR-based Kalman filter and IE across five brain rhythms in EEG, the IF and IE of the EOG channel, the raw EEG spectrogram, and the corresponding hypnogram are shown in Figure 3. Due to the bandpass filtering stage, all estimated IFs are confined within their respective bandwidths. Visual inspection of this sample record suggests a strong correlation between the frequency variations of the hypnogram and the IF variations. This observation is further investigated across all subjects and records.

**Figure 5:**
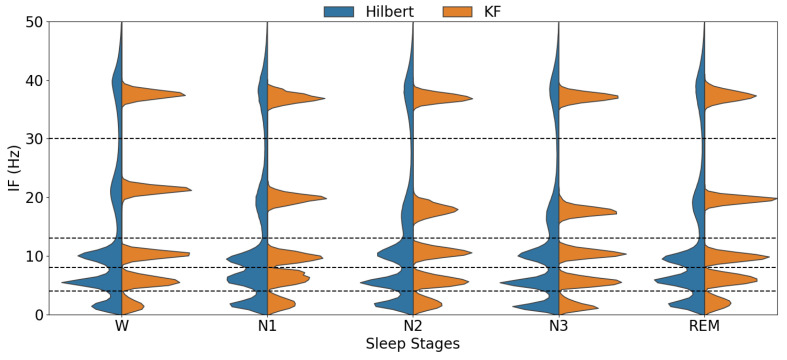
Sample distribution comparison of the IF for different EEG sub-bands (from bottom to top: δ, θ, α, β and γ, separated by dashed lines) per wake/sleep stages obtained via Hilbert transform (in blue) vs. the proposed KF-based approach (in orange) for record SC4001 of Sleep-EDFx. The KF-based method demonstrates substantially reduced variance in IF estimation compared to the Hilbert transform, as evidenced by the more compact distributions. This enhanced precision improves the differentiation between sleep stages, particularly in the beta frequency band, where the distributions show a clear separation across wake/sleep states.

A comparison between the conventional Hilbert transform-based method and the KS approach for estimating IF on real data are made in Figure 5. This figure shows the distribution of the IF across different frequency bands per sleep stage for one of the recordings (SC4001 of Sleep-EDFx). The comparison is made between the raw IF values extracted using the Hilbert transform and those obtained using the KS without any averaging. Accordingly, the IF estimates derived from the Hilbert Transform do not significantly vary between different sleep stages, showing substantial overlap in their distributions across different sleep stages. In addition, the Hilbert estimated IF has a high deviation in beta and gamma bands, while our proposed Kalman smoother-based approach has a significantly lower variance in these upper frequencies, resulting in more precise and reliable frequency tracking. Finally, the IF distributions obtained using KS are visually more distinct, particularly in the beta and gamma bands, where they show visible differences between various sleep stages.

As shown in Figure 5, the mean value of the beta IF during the REM stage is similar to the frequencies observed during wakefulness and the N1 stage. This observation aligns with the known physiological characteristics of REM sleep.

### 4.2. Classification Results Across All Subjects

**Figure 6:**
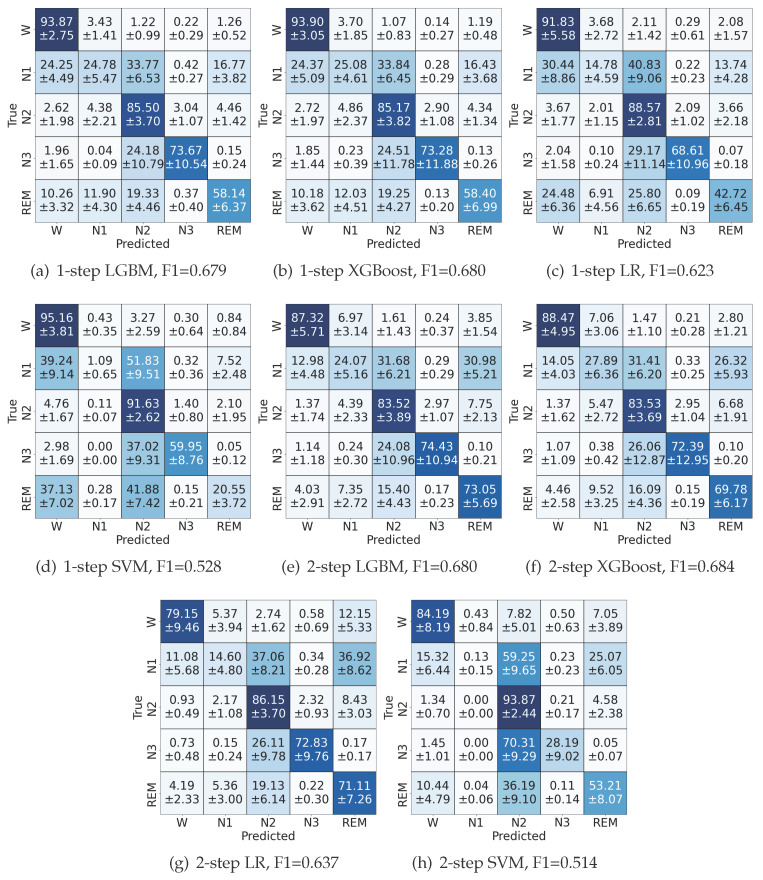
Confusion matrices of the top-performing 1-step and 2-step classifiers with their respective F1 scores on Sleep-EDFx. A subject-wise 10-fold cross-validation was employed, with eight subjects in each test fold, ensuring each subject appeared in a test fold exactly once. For each fold, a separate confusion matrix was calculated. Then, the average and standard deviations for each entry were calculated across all 10 folds. The 2-step classifiers achieved higher accuracy for the REM stage, with a slight decrease in performance for the wake and N2 periods. (**a**) 1-step LGBM, F1 = 0.679, (**b**) 1-step XGBoost, F1 = 0.680, (**c**) 1-step LR, F1 = 0.623, (**d**) 1-step SVM, F1 = 0.528, (**e**) 2-step LGBM, F1 = 0.680, (**f**) 2-step XGBoost, F1 = 0.684, (**g**) 2-step LR, F1 = 0.637, (**h**) 2-step SVM, F1 = 0.514.

The sleep stage classification results across all data records using the described methods are summarized in the confusion matrices of Figure 6.

Overall, the LGBM and XGB classifiers demonstrated the highest performances among all tested classifiers, achieving an F1-macro score of 0.69, Cohen’s Kappa of 0.68, and an accuracy of 77%. In terms of individual sleep stages, the wakefulness state showed the highest classification performance, while N1 stage exhibited frequent misclassifications with wakefulness, N2, and REM stages. N2 and N3 stages, along with REM stage, were most effectively detected using the LGBM model.

The confusion matrices indicate that Gradient Boosting methods, such as XGBoost and LGBM, exhibit higher overall mean accuracy compared to other classifiers with the proposed feature vector.

The comparison between one-step and two-step LGBM classifiers shows a significant trade-off in performance. While the two-step approach substantially improved REM sleep detection accuracy from 58% to 73%, it also led to a decline in wakefulness detection accuracy from 94% to 87%. Throughout this transition, the detection accuracy of non-REM sleep stages remained largely consistent between both approaches.

A detailed analysis of sensitivity (SE) and specificity (SP) for each sleep stage is presented in Table 1. Both one-step and two-step classification approaches show distinct patterns across different sleep stages. The two-step classification approach notably improved wake detection sensitivity (from 86% to 93% for XGBoost), but showed a slight decrease in specificity. While N2 and N3 stages maintained relatively stable detection rates across both approaches, the N1 stage proved particularly challenging with the lowest sensitivity (34–38%) and specificity (24–28%) across all configurations. This difficulty in N1 classification aligns with the known challenges in distinguishing transitional sleep stages. The REM stage showed an interesting trade-off between approaches; while the one-step classification achieved higher sensitivity (around 68%), the two-step approach improved specificity, particularly for LGBM (from 58% to 73%). These results suggest that different classification approaches (or a combination of multiple classifiers) might be optimal depending on the specific sleep stages of interest.

### 4.3. Subject-Wise Performances

The classifiers’ effectiveness was also evaluated through subject-wise analysis. We evaluated performances using two widely adopted metrics in the literature: Cohen’s kappa (κ) and the macro-averaged F1 score. Table 2 presents the average performance metrics across all subjects. The boosting models, LGBM and XGBoost, demonstrated superior performance with 77% accuracy, an F1-score of 0.69, and a Cohen’s kappa of 0.68.

**Figure 7:**
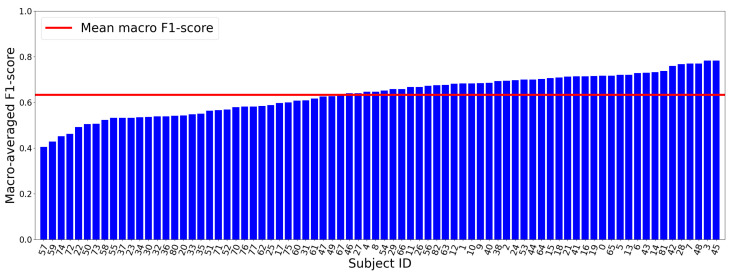
Sorted macro-averaged F1-scores across subjects using LGBM classifier. 50% of subjects achieved an F1-score above 0.62.

Further analysis of model performance across subjects is illustrated by the macro-averaged F1-score in Figure 7 using LGBM classifier. Subject 7 achieved the highest macro-averaged F1-score of 0.77, while subject 74 recorded the lowest at 0.37. Notably, over 50% of the subjects obtained a macro-averaged F1-score exceeding 0.6, underscoring the model’s effectiveness in differentiating between various sleep stages reliably across different subjects.

### 4.4. Feature Importance

**Figure 8:**
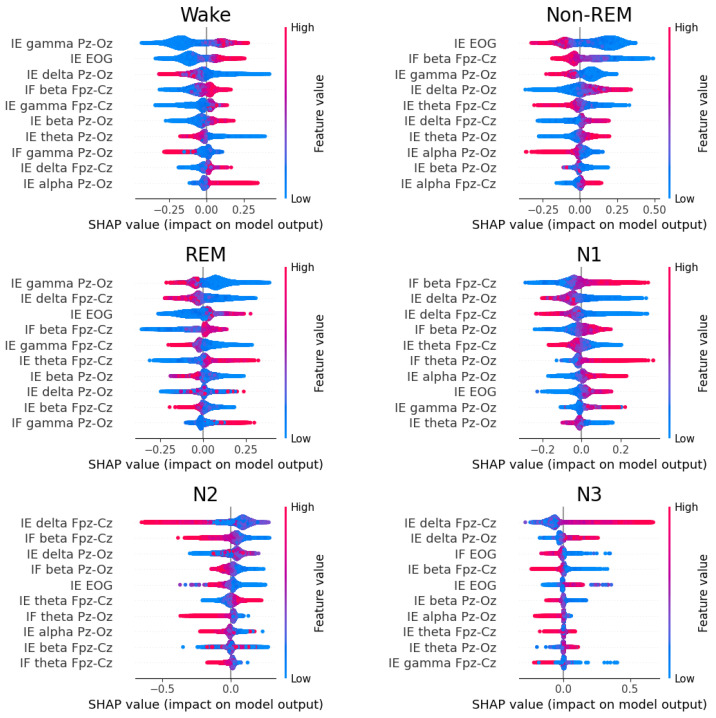
Shapley values for each class at every stage of the two-step classifier, highlighting the top 10 important features. Features γ, β, and δ emerge as the most discriminative across wake, REM, and non-REM stages.

We used SHAP values to assess the significance of each feature in the model’s decision-making process and to understand how EEG characteristics contribute to sleep stage classification. Our analysis was conducted on the entire population rather than subject-wise, providing a robust, population-level understanding of feature importance. Figure 8 presents the Beeswarm plots of the SHAP values for the LGBM model, showing the top ten important features for each class, computed on each fold of the test dataset using the one-vs-rest (OvR) approach.

The SHAP analysis revealed patterns that align well with established sleep physiology literature, particularly regarding the roles of γ, β, and δ activities across different sleep stages. As shown in Figure 8, γ activity plays a crucial role in distinguishing sleep stages from wakefulness, being prominent during wakefulness and diminishing during sleep. In REM sleep, β activity is notably higher compared to non-REM sleep, though the SHAP values for the N1 stage are very close to those for REM, explaining the frequent misclassification between these stages. The N2 and N3 sleep stages showed smaller SHAP values for β-band features, indicating reduced β activity during these deeper sleep phases. For the N3 stage specifically, Shapley analysis highlighted elevated values for δ features and lower β values compared to N1 and N2, reflecting the predominance of low-frequency brain activity during the deepest sleep stages [73]. EOG-derived features showed expected patterns of importance, particularly in distinguishing between wake and REM states. EOG activity is elevated during wakefulness and REM sleep, while non-REM stages are associated with lower SHAP values for EOG features. Among non-REM stages, N1 shows higher SHAP values, indicating greater EOG activity, which aligns with common understanding of N1 characteristics. The strong alignment between our population-level SHAP analysis and previous physiological findings suggests that these features capture fundamental aspects of sleep stage transitions that are generally consistent across individuals.

## 5. Discussion

The findings of this study demonstrate the effectiveness of our proposed KF-based approach for tracking instantaneous frequency (IF) in EEG signals during different sleep stages. This method offers specific advantages in addressing critical challenges in EEG frequency tracking and provides interpretable insights into the associated physiological processes, as discussed below.

### 5.1. Enhanced Frequency Tracking

Our KF-based approach has a key advantage over the conventional Hilbert transform-based technique by producing notably more narrow IF distributions (lower estimation variance) compared to the Hilbert transform, which has a high variance and is particularly susceptible to background cerebral activity. Our KF-based scheme improved precision in IF estimation, enabling better differentiation between sleep stages, especially evident in the beta–gamma frequency bands where distinct separations between wake/sleep states are observed in our classification results.

### 5.2. Pipeline Validation Through Sleep Stage Analysis

The effectiveness of our IF tracking approach was validated through its application to sleep stage classification. The two-step LGBM classifier demonstrated strong performances, achieving an F1-macro score of 0.69 and Cohen’s Kappa of 0.68, with an overall accuracy of 77%. These results are particularly noteworthy given the challenging nature of sleep stage classification and the minimal feature set employed in this study. The SHAP value analysis provides additional validation of our approach’s interpretability and physiological relevance. The feature importance rankings align remarkably well with established literature on sleep physiology, demonstrating that our IF tracking method successfully captures physiologically meaningful frequency variations in the EEG.

### 5.3. Limitations

Despite the promising results, several limitations should be acknowledged. First, while our frequency tracking performance shows significant improvements over traditional methods, the accuracy of classification for certain sleep stages, particularly N1, remains challenging. This limitation reflects the inherent difficulty in distinguishing transitional sleep stages, even for human experts.

A second limitation of the current implementation is its reliance on conventional machine learning classifiers. Although these classifiers offer good interpretability, more advanced sequential architectures, such as recurrent neural networks (RNNs), long short-term memory (LSTM), or other recurrent deep neural networks, could better capture temporal dependencies and potentially enhance classification performance. The rationale for adopting the more classical architectures was to maintain the focus of this work on establishing the Kalman filtering framework for EEG frequency tracking. Nonetheless, relying solely on instantaneous frequency is unlikely to capture the complexities of sleep staging; combining hybrid features with multiple classification schemes is anticipated to yield superior performance over any single approach.

Another limitation is that the study did not implement specific artifact removal techniques to address common sleep-related artifacts, such as motion artifacts from rolling movements, changes in electrode impedance, saccades and electrooculogram artifacts. While our method demonstrated good performance on the available dataset, the impact of these artifacts on its robustness needs to be systematically evaluated in future work. While keeping the preprocessing minimal to preserve the intricacies of EEG data [74], incorporating more advanced EEG preprocessing techniques and artifact removal methods, such as independent component analysis [75,76] or nonstationary component analysis [77], is expected to improve the accuracy of EEG instantaneous frequency tracking.

Additionally, the generalizability of our findings presents another limitation. Although our population-level SHAP analysis provided insights into feature importance that align with established sleep physiology, the study was conducted on a specific dataset under controlled recording conditions. Future studies should explore subject-specific feature importance patterns and validate the method across diverse populations, including individuals with specific sleep disorders, different age groups, and varying recording environments.

### 5.4. Future Directions

The proposed methodology has several implications for consciousness analysis and offers opportunities for future research. Some of the key areas for further exploration are outlined below.

#### 5.4.1. IF Estimate Confidence Interval

**Figure 9:**
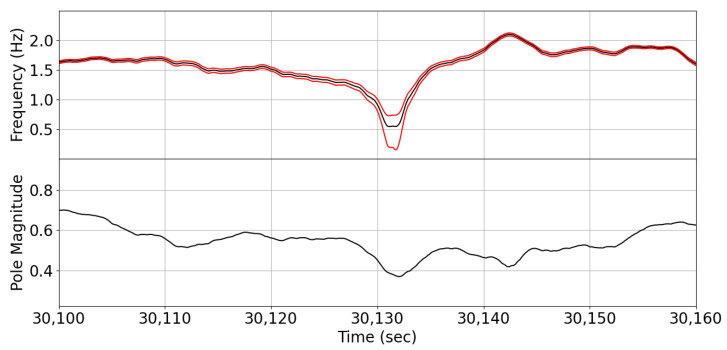
(**Top**) The estimated instantaneous frequency (IF) (black line) with its ± standard deviation confidence intervals (red lines) derived from subject SC4001 of the Sleep-EDFx dataset; (**bottom**) the AR model pole magnitude. The uncertainty in IF estimation decreases as pole magnitude approaches unity, indicating dominant oscillatory behavior with potential sleep-related physiological interpretations.

Kalman filters have inherent mechanisms for confidence interval assessment. To assess the confidence of the estimated IF values, we monitored the lower and upper bounds defined by the intervals $\left[\mathrm{IF}\left(n\right)-\sigma_{\mathrm{IF}}\left(n\right),\mathrm{IF}\left(n\right)+\sigma_{\mathrm{IF}}\left(n\right)\right]$, where $\sigma_{\mathrm{IF}}$ represents the standard deviation of the estimated values (obtained from the KF covariance matrix estimates). For example, Figure 9 shows the estimated ${\mathrm{IF}}_{\delta }$ along with its confidence interval and the magnitude of the corresponding AR model pole for a small segment of the EEG data. As the pole magnitude approaches the unit circle, the uncertainty in the estimated IF decreases. This is consistent with the fact that for a narrow-band signal when the generating model pole magnitudes approach the unit circle, the system output becomes more oscillatory. Therefore, the quality factor (Q-factor) of the EEG is increasing, making estimated IF more reliable [42]. In narrow-band EEG signals, an oscillatory instantaneous pole can potentially provide insights into spontaneous or event-related physiological activity. Furthermore, in multichannel EEG analysis, this measure could assist in localizing the sources of oscillatory frequencies.

#### 5.4.2. Drowsiness Detection

**Figure 10:**
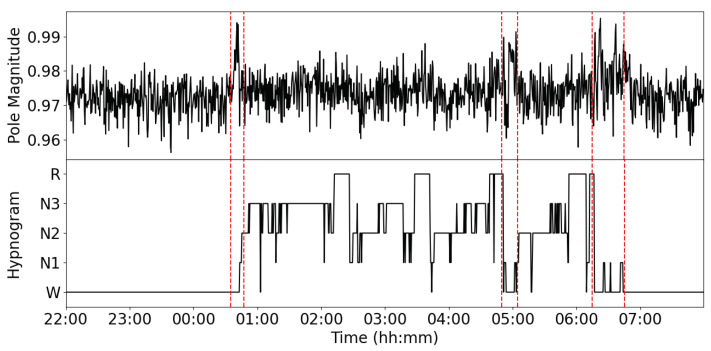
Instantaneous pole magnitude of the α band and corresponding hypnogram label for record SC4001 of Sleep-EDFx. Pole magnitude approaching the unit circle during sleep–wake transitions (denoted by the short segments between red dashed lines) suggests it can serve as a marker for drowsiness detection.

Aside from sleep stage analysis, the proposed method has promising applications in drowsiness detection. By examining AR model pole-magnitude variations along the hypnogram, distinct behaviors can be observed during transitions between wakefulness and sleep. For instance, the α-wave AR model pole-magnitude extracted from the occipital region channel shows notable changes before the onset of sleep. Figure 10 illustrates a typical case from the Pz–Oz channel, where the pole amplitude approaches one just before the sleep onset and returns to its baseline value after the onset of sleep. This transient oscillatory behavior is also evident during the sleep–wake transition.

Given the prominent presence of the α rhythm during sleep [78], the observed phenomenon can be interpreted as indicative of transitions between wakefulness and sleep or as a sign of drowsiness. Monitoring drowsiness is crucial for applications that require alertness detection, such as for drivers, pilots, and control center operators. However, commonly used datasets, like those in this study, lack drowsiness-specific labels in the hypnograms. Consequently, testing this hypothesis fully would require new datasets and recording setups designed to capture drowsiness states explicitly.

#### 5.4.3. Kalman Filter Versus Kalman Smoother

In this study, data were processed offline using a *fixed-interval* Kalman smoother to track the AR model poles. For online applications, a *fixed-lag* Kalman smoother (e.g., with a delay of ten to thirty seconds) can be employed. This extension would provide the benefits of Kalman smoothing for real-time sleep staging.

#### 5.4.4. Comparison with Existing Methods and Broader Applications

The primary objective of this study was to develop and validate a robust Kalman filter-based framework for tracking instantaneous frequency in EEG signals during different sleep stages. While our results demonstrate the effectiveness of the frequency-tracking pipeline in sleep staging, the method’s utility extends beyond sleep analysis. This robust frequency-tracking framework could prove valuable for a wide range of EEG applications where frequency components are key features, such as brain–computer interfaces, seizure detection, emotion recognition, and cognitive state assessment. Future work could involve comprehensive comparisons with other EEG-based features, existing sleep stage classification schemes and applications of this framework to other EEG analysis domains. Such studies would quantitatively assess the method’s advantages in terms of accuracy, robustness to artifacts, and computational efficiency across various applications.

## 6. Conclusions

This study introduced a robust Kalman filtering framework for tracking instantaneous frequency in EEG data, providing more stable and accurate estimates than traditional methods such as the Hilbert transform. The effectiveness of this approach was highlighted through its application to sleep stage classification, where the extracted frequency features enabled accurate differentiation between sleep stages. The two-step classification strategy achieved 76.57% accuracy and a Cohen’s kappa of 0.677, validating the reliability of our frequency tracking method. Interpretability, assessed via SHAP value analysis, indicated that the tracked frequency components aligned closely with established neurophysiological patterns across different brain states, underscoring the physiological relevance and explainability of this approach. The demonstrated accuracy and stability of this framework potentially pave the way for precise frequency tracking in other neurophysiological applications where characterizing the time-varying spectral content of the EEG is essential.

## Figures and Tables

**Table 1:** Sensitivity (SE) and Specificity (SP) comparisons for XGBoost and LGBM classifiers across different sleep stages. Results show mean ± standard deviation from 10 to fold cross-validation. #Step indicates one-step (direct classification into 5 stages) versus two-step classification (first into Wake/Non-REM/REM, then Non-REM into N1/N2/N3). Both classifiers show the highest performance for Wake stage detection and the lowest for N1 stage.

Model	#Step	Metric	Wake	N1	N2	N3	REM
XGBoost	1	SE	86.08±3.68	36.87±4.81	78.97±4.35	81.19±8.20	68.47±6.00
		SP	93.90±3.05	25.08±4.61	85.17±3.82	73.28±11.88	58.40±6.99
LGBM	1	SE	86.10±3.73	38.01±5.08	78.94±4.59	78.65±8.79	67.83±6.28
		SP	93.87±2.75	24.78±5.47	85.50±3.70	73.67±10.54	58.14±6.37
XGBoost	2	SE	93.09±1.48	34.50±6.15	79.64±4.73	80.68±8.48	61.20±6.02
		SP	88.47±4.95	27.89±6.36	83.53±3.69	72.39±12.95	69.78±6.17
LGBM	2	SE	92.28±3.41	34.33±6.45	79.90±4.97	80.59±8.69	57.81±6.22
		SP	87.32±5.71	24.07±5.16	83.52±3.89	74.43±10.94	73.05±5.69

**Table 2:** Performance comparison of different classifiers for sleep stage classification. Results show F1-macro score, Cohen’s kappa, and accuracy (mean ± standard deviation) across 10-fold cross-validation. #Step indicates whether classification was performed in one or two stages. LGBM and XGBoost (shown in bold) achieved the best performance across all metrics.

Model	#Step	F1-Macro	Cohen’s Kappa	Accuracy
SVM	1	0.528±0.024	0.578±0.004	71.34±2.59
LR	1	0.623±0.028	0.633±0.040	74.33±2.88
**XGBoost **	1	0.680±0.028	0.683±0.045	77.40±3.27
**LGBM**	1	0.679±0.028	0.684±0.044	77.45±3.12
SVM	2	0.514±0.041	0.574±0.056	70.60±4.31
LR	2	0.637±0.026	0.633±0.051	73.33±4.05
**XGBoost**	2	0.684±0.029	0.680±0.045	76.82±3.32
**LGBM**	2	0.680±0.028	0.677±0.048	76.57±3.61

## Data Availability

Data is publicly available only.

## Abbreviations

AASM	American Academy of Sleep Medicine	KF	Kalman filter
AR	Auto-Regressive	KS	Kalman Smoother
ARMA	Auto-Regressive Moving Average	LGBM	Light Gradient Boosting Machine
CV	Cross-Validation	LR	Logistic Regression
DOA	Depth of anesthesia	LSTM	Long Short-Term Memory
EEG	Electroencephalogram	MMSE	Minimum Mean Square Error
EOG	Electrooculogram	OvR	One-vs-Rest
FIR	Finite Impulse Response	PSD	Power Spectral Density
IE	Instantaneous Energy	PSG	Polysomnography
IF	Instantaneous Frequency	REM	Rapid Eye Movement
IP	Instantaneous Phase	RNN	Recurrent Neural Network
R&K	Rechtschaffen and Kales	SHAP	Shapley Additive Explanations
STFT	Short-Time Fourier Transform	SVM	Support Vector Machine
TVAR	Time-Varying Auto-Regressive	WGN	White Gaussian Noise
XGB	Extreme Gradient Boosting		
